# Infection prevention practices in the Netherlands: results from a National Survey

**DOI:** 10.1186/s13756-019-0667-3

**Published:** 2020-01-06

**Authors:** Anita Huis, Jeroen Schouten, Dominique Lescure, Sarah Krein, David Ratz, Sanjay Saint, Marlies Hulscher, M. Todd Greene

**Affiliations:** 1 Radboud Institute for Health Sciences, IQ healthcare, Radboud University Medical Center, PO box 9101 (114), 6500 HB Nijmegen, The Netherlands; 2 Erasmus MC, University Medical Center Rotterdam, PO box 2040, 3000 CA Rotterdam, The Netherlands; 3 VA Ann Arbor Center for Clinical Management Research, VA Ann Arbor Healthcare System, 2800 Plymouth Road, North Campus Research Complex 16, Ann Arbor, MI 48109 USA; 4 Department of Internal Medicine, University of Michigan Medical School, Ann Arbor, USA; 5 VA/UM Patient Safety Enhancement Program, Ann Arbor, USA

**Keywords:** Infection control, Healthcare-associated infection, Nosocomial, Hospitals, Implementation

## Abstract

**Objective:**

To examine the extent to which acute care hospitals in the Netherlands have adopted recommended practices to prevent catheter-associated urinary tract infection (CAUTI), central line-associated bloodstream infection (CLABSI), ventilator-associated pneumonia (VAP), and *Clostridioides difficile* infection (CDI).

**Methods:**

Between 18 July 2017 and 31 October 2017, we surveyed the infection prevention teams of all acute care hospitals in the Netherlands. The survey instrument was based on the ‘Translating Healthcare-Associated Infection Prevention Research into Practice’ (TRIP) questionnaire and adapted to the Dutch context. Descriptive statistics were used to examine the reported regular use of CAUTI, CLABSI, VAP, and CDI prevention practices as well as the hospital characteristics.

**Results:**

Out of 72 eligible hospitals, 47 (65.3%) responded. Surveillance systems for monitoring CAUTI, CLABSI, VAP, and CDI were present in 17.8, 95.4, 26.2, and 77.3% of hospitals, respectively. Antimicrobial stewardship programs have been established in 91.5% of participating hospitals. For CAUTI, the majority of hospitals regularly used aseptic technique during catheter insertion (95%) and portable bladder ultrasound scanners (86.1%). Intermittent catheterization and catheter stop-orders were regularly used by 65.8 and 62.2% of hospitals. For CLABSI, all hospitals regularly used maximum sterile barrier precautions and chlorhexidine gluconate for insertion site antisepsis. Avoidance of the femoral site for central line insertions was regularly used by 65.9% of hospitals. Urinary catheters and central-lines impregnated with antibiotics or antiseptics were rarely used (≤ 5%). Selective decontamination strategies for preventing VAP were used in 84% of hospitals. With the exception of disposable thermometers (31.8%), all prevention practices to prevent CDI were regularly used by more than 80% of hospitals.

**Conclusions:**

Most Dutch hospitals report regular use of recommended practices for preventing CLABSI and CDI. Several specific practices to prevent CAUTI and VAP were less frequently used, however, providing an opportunity for improvement.

## Background

Healthcare-associated infection (HAI) is a serious and persistent problem throughout the world. HAIs are burdensome to patients, complicate treatment, prolong hospital length-of-stay, induce resistance of microorganisms to antimicrobials, raise healthcare costs, and can be life-threatening [1–5]. In 2011, the estimated prevalence of HAIs in European acute care hospitals was 5.7%, affecting approximately 3.2 million patients [6]. The most commonly reported infection types were respiratory tract infections (23.5%), surgical site infections (19.6%), urinary tract infections (19%), bloodstream infections (10.7%), and gastro-intestinal infections (7.7%), of which 48% were *Clostridioides difficile* infection (CDI) [6]. Hospital surveillance in the Netherlands showed a decreasing prevalence of HAIs from 7.2% in 2008 [7] to 5.0% in 2017 [8]. Despite this decrease, point prevalence of HAIs varies considerably between Dutch hospitals, ranging from 1.5 to 6.5% [8]. The distribution of hospitals, the severity of patient case mix or infection types did not seem to explain the differences between the hospitals [8].

Major types of HAIs are device-associated, including catheter-associated urinary tract infection (CAUTI), central line-associated bloodstream infection (CLABSI), and ventilator-associated pneumonia (VAP) [9–11]. The Dutch surveillance data of 2017 pointed out that 17% of the observed HAIs were symptomatic urinary tract infections - of which 62% were catheter-associated. Primary bloodstream infections accounted for 0.8% of the observed HAIs of which 33% were central line-associated, and pneumonia accounted for 20% of the observed HAIs - of which 21% were ventilator-associated [8].

It is estimated that 65% of CAUTIs, 55% of CLABSIs, and 55% of VAPs could be prevented by using certain evidence-based infection prevention strategies [12, 13]. Most strategies focus on a core set of recommended prevention practices, based on guidelines from numerous professional organizations and government agencies [14–17]. The extent to which these practices are adopted by Dutch hospitals is unknown. We thus conducted a nationwide cross-sectional survey to evaluate the use of currently recommended practices for preventing CAUTI, CLABSI, VAP, and CDI in the Netherlands.

## Methods

### Study design and survey instrument

Data for this cross-sectional study were collected using a survey instrument based on the ‘Translating Healthcare-Associated Infection Prevention Research into Practice’ (TRIP) questionnaire developed by Krein, Saint, and colleagues and previously distributed in the USA, Japan, and Thailand [18–24]. The instrument contained questions about general hospital characteristics (e.g. number of beds), general infection prevention policies (e.g. presence of guidelines and surveillance systems), staffing of the infection control program, and use of specific practices related to the prevention and monitoring of CAUTI, CLABSI, VAP, and CDI. The survey instrument was forward-backward translated into Dutch by the research team with support of a bilingual translator and digitalized afterwards with LimeSurvey. Some questions were slightly modified to fit the Dutch context. These questions mainly related to hospital characteristics, job functions, and organizational structure. As in the original survey instrument, respondents were asked to indicate the frequency of use for certain infection prevention practices on a scale from 1 (never use) to 5 (always use). The survey instrument was pilot tested by five infection preventionists from five different hospitals, recruited via the Dutch Association for Hygiene & Infection Prevention in Healthcare, to ensure the validity, reliability, and acceptability of the survey. No changes were made after pilot testing (see Additional file 1).

### Data collection procedure

Between July 18, 2017 and October 31, 2017, we approached 72 infection prevention teams, representing all acute care hospitals in the Netherlands, to participate in the survey. We first contacted infection prevention teams by telephone to explore their willingness to participate. An email invitation with log-in details was then sent to the infection prevention representative of the teams that agreed to participate. A reminder e-mail was sent after 2 weeks to non-responders. After 4 weeks, those who had not yet responded were contacted by telephone. The survey responses were anonymized**.**

### Statistical analysis

Descriptive statistics, n (%) for categorical and mean ± standard deviation (SD) for continuous variables were examined for all hospital characteristics as well as specific infection prevention practices. Responses about practice use were further categorized, with responses of 4 or 5 (i.e. ‘almost always use’ or ‘always use’) defined as regular use and coded as 1, and 0 otherwise. All statistical analyses were conducted in SAS V9.4 (Cary, NC).

## Results

Infection prevention teams of 47 hospitals completed the questionnaire, yielding a response rate of 65% (47/72). Nineteen out of the 72 hospitals declined to participate in advance, mainly because of time constraints. Of these, three hospitals explicitly indicated that they had not implemented specific infection prevention measures with regard to CAUTI, CLABSI, VAP, and CDI. Of the 53 hospitals that were willing to participate in the survey, 6 did not complete the questionnaire, despite several reminders.

Select hospital characteristics are shown in Table 1. The responding hospitals averaged 514.1 acute care beds (median 481) with 21.0 Intensive Care Unit (ICU) beds (median 12). There was an average of 4.8 infection prevention personnel, with an average of 3.9 Full-Time Equivalent (FTE) employees, at the hospitals. All respondents indicated that their hospital had clear infection prevention guidelines present that support the clinical teams, and 91.5% indicated that all the resources and materials for appropriate application of the guidelines were available. An antimicrobial stewardship program was present in 43 (91.5%) hospitals.

**Table 1:** Hospital characteristics

Characteristic	n (%)/mean ± sd
Total number of acute care hospital beds (including ICU beds)	514.1 ± 260.1
Range	140–1100
Total number of adult ICU beds	21.0 ± 18.7
Range	5–81
Percentage of multiple bed rooms	66.4 ± 22.1
Number of infection prevention experts	4.8 ± 1.9
Number of FTE for the infection prevention experts	3.9 ± 1.8
Number of beds per FTE of infection prevention experts	131.2 ± 48.6
Range	48.5–291.2
Number of medical microbiologists	2.9 ± 1.9
Number of FTE for the medical microbiologists	2.0 ± 1.9
Hospital cooperates with other hospitals or agencies with regard to stimulating infection prevention	30/46 (65.2%)
Clear infection-prevention guidelines present that support caregivers	47/47 (100%)
Resources and materials available for proper observance of the infection-prevention guidelines	43/47 (91.5%)
Good to very good support of the hospital management infection-prevention policy	41/47 (87.2%)
System for monitoring the number of patients who have urinary catheters placed	9/45 (20.0%)
System to monitor how long the patient has a urinary catheter	9/45 (20.0%)
Established* surveillance system for monitoring CAUTI rates	8/45 (17.8%)
Daily check whether the presence of a central venous catheter is still indicated	39/43 (90.7%)
Established* surveillance system for monitoring CLABSI rates	41/43 (95.4%)
Established* surveillance system for monitoring VAP rates	11/42 (26.2%)
Protocol for routinely testing for CDI as soon as patients develop diarrhea	23/44 (52.3%)
Surveillance system for monitoring the number of patients with CDI	34/44 (77.3%)
Presence of an antimicrobial stewardship program	43/47 (91.5%)

* Case definitions and surveillance methods used are embedded within the Dutch PREZIES network for the surveillance of hospital acquired infections and based on the surveillance protocols of the European Centre for Disease Prevention and Control. *Abbreviations*: *ICU* Intensive care unit, *FTE* Full time equivalent, *CAUTI* Catheter-associated urinary tract infection, *CLABSI* Central line-associated bloodstream infection, *VAP* Ventilator-associated pneumonia, *CDI Clostridoides difficile* infection

The presence of surveillance systems for monitoring infection rates varied considerably by infection type. Surveillance for monitoring CAUTI rates was the lowest, with only 17.8% of hospitals with an established system. Additionally, only 20% of hospitals had established systems to monitor urinary catheter placement and duration. Conversely, nearly all hospitals used an established surveillance system for monitoring CLABSI rates (95.4%), and the vast majority performed a daily check for whether the presence of a central venous catheter was still indicated (90.7%). Surveillance systems for monitoring VAP rates were present in only 26.2% of hospitals. A surveillance system for monitoring the number of patients with CDI was reported by 77.3% of the participating hospitals.

**Fig. 1:**
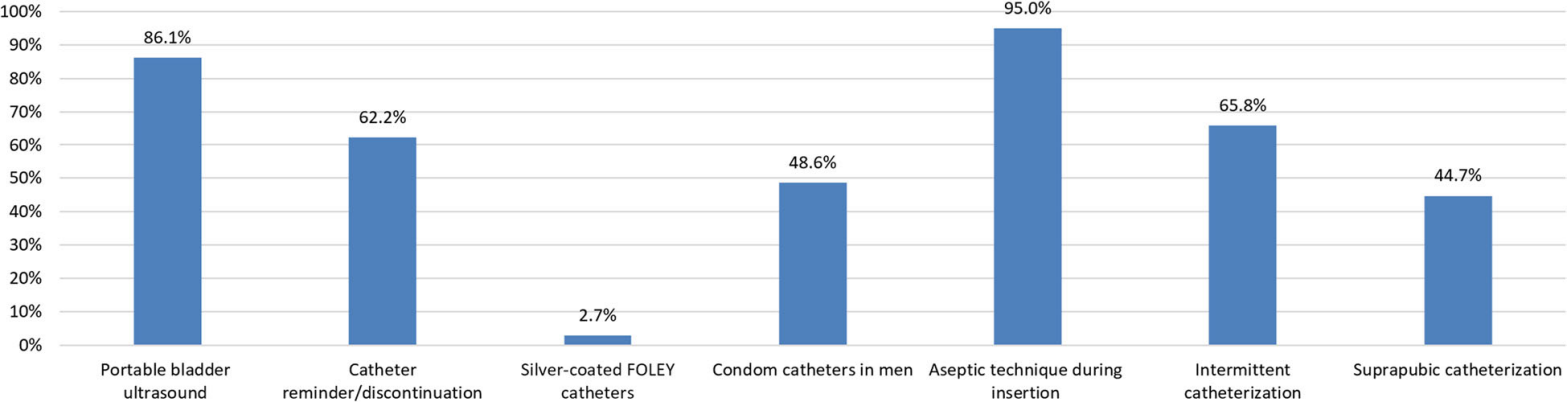
Reported regular use of CAUTI prevention practices

**Fig. 2:**
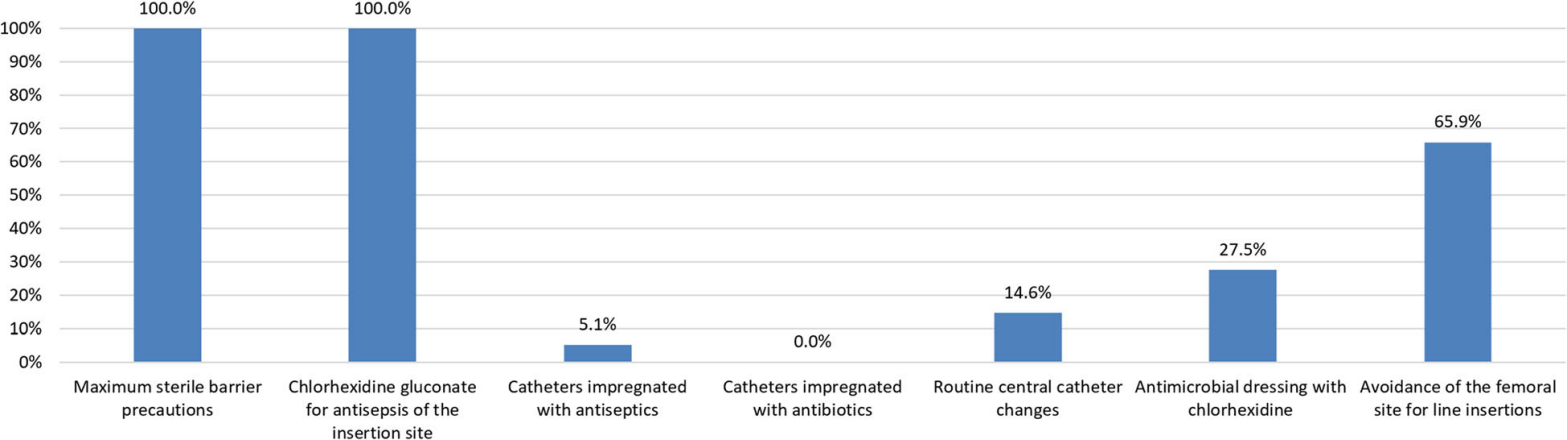
Reported regular use of CLABSI prevention practices

**Fig. 3:**
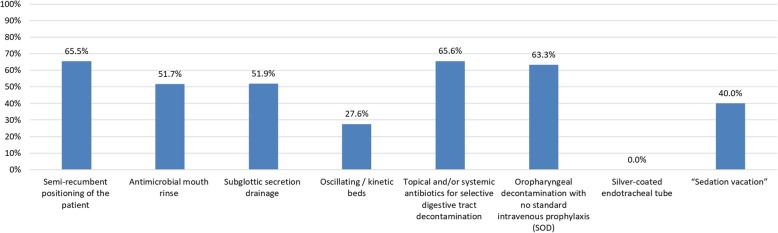
Reported regular use of VAP prevention practices

**Fig. 4:**
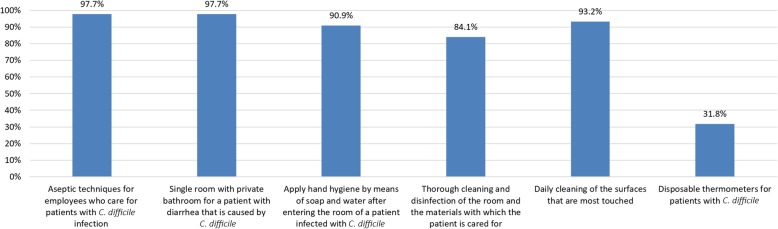
Reported regular use of CDI prevention practices

The regular use of Infection prevention practices specific to CAUTI, CLABSI, VAP, and CDI are displayed in Figs. 1, 2, 3 and 4. Several CAUTI infection prevention practices (Fig. 1) were regularly used by the majority of responding hospitals, including: aseptic technique during insertion (95.0%), and portable bladder ultrasound scanners (86.1%). Intermittent catheterization and catheter reminder/nurse initiated discontinuation were regularly used by 65.8 and 62.2% of the hospitals, respectively. Two CLABSI prevention practices (Fig. 2) were regularly used by all responding hospitals: maximum sterile barrier precautions and chlorhexidine gluconate for antisepsis of the insertion site. Avoidance of the femoral site for central line insertion was regularly used in 65.9% of responding hospitals. Urinary catheters and central venous catheters impregnated with antiseptics were seldomly used (5.1%), and central venous catheters impregnated with antibiotics were not used at all. Three VAP prevention practices (Fig. 3:) were used regularly by more than 60% of the responding hospitals including: semi-recumbent positioning (65.5%), topical and systemic antibiotics for selective digestive tract decontamination (65.6%) and oropharyngeal decontamination (63.3%). Finally, all but one CDI prevention practice (Fig. 4) were used by over 80% of responding hospitals.

## Discussion

Over the last decade, preventing HAIs has been a major priority worldwide and in the Netherlands [25, 26]. Implementing infection prevention measures is vital to preventing HAIs [12, 13, 27]. Knowing which recommended practices are currently used and to what extent is a starting point for the development of effective strategies for improving infection prevention efforts [28]. Several important findings emerged from our nationwide Dutch study.

First, systems for routinely monitoring CAUTI rates, as well as the placement and the duration of urinary catheters in hospitalized patients are only present in approximately 20% of the hospitals, despite the link between urinary catheters and subsequent infection [29]. The number of hospitals that regularly use urinary catheter reminders or stop-orders and/or nurse initiated catheter discontinuation to prompt timely catheter removal is much lower (62%) than a similar process for CLABSI prevention involving the use of daily checks for whether a central line is still indicated (90%). A major difference between the prevention of CAUTI and CLABSI is that the majority of patients with central lines are admitted to an ICU. Implementing urinary catheter reminders or stop-orders hospital-wide is generally more challenging than within a specific ward. Institutional use of CAUTI prevention practices may be achieved through a hospital-wide approach focused on implementing a bundle of CAUTI preventive practices [30, 31].

Second, more than 90% of the hospitals monitored CLABSI and performed a daily check whether the presence of a central line was still indicated. This may be a reason why we observed rare use of routine central line changes in Dutch hospitals. In the Netherlands, monitoring of CLABSI is facilitated by a well-established nationwide surveillance system [32], using strict criteria for assessing CLABSI based on the definitions from the European Centre for Disease Control [33]. Since 2009, CLABSI incidence in ICUs decreased from 4.3/1000 central line days to 1.2/1000 central line days in 2016 [34, 35]. It is likely that the national patient safety program ‘Prevent harm, work safely’ has contributed to this decline. A key focus of this five-year programme (2008–2012) was on the implementation of a central-line insertion and maintenance bundle [36]. The programme demonstrated that the compliance rate for the bundle element ‘Daily check whether the central line is still indicated’, increased from 60% to ≥80% in 2014 [37]. A recent meta-analysis by Ista and colleagues showed that the incidence of CLABSI decreased significantly after the implementation of central-line bundles, from 6.4/1000 catheter days to 2.5/ 1000 catheter days [38]. A significant proportion of patients with a central venous catheter are admitted to an ICU, and many CLABSI prevention practices have primarily been implemented in ICUs. This is reflected in the Dutch surveillance data of CLABSI incidence in non-ICUs. Between 2009 and 2016, only a small decrease in CLABSI incidence occurred within non-ICUs (from 3.5/1000 line days to 3.3/1000 line days) [34, 35]. This finding underscores the importance of also effectively implementing CLABSI infection practices outside of the ICU setting.

Third, VAP rates are rarely monitored by Dutch hospitals. This is probably due to the ongoing debate about which definition and criteria should be used for this complication [39, 40]. While the diagnosis of VAP is usually made on clinical, microbiological and radiological criteria, low interrater reliability and poor correlation with histopathology have been described [39, 41]. A meta-analysis of three Dutch studies on the effects of systemic antibiotics for selective digestive tract decontamination and oropharyngeal decontamination showed that both practices reduce ICU mortality and ICU-acquired Gram-negative bacteraemia, without increasing antibiotic resistance [42, 43]. Based upon these findings, the use of systemic antibiotics for selective digestive tract decontamination and oropharyngeal decontamination in patients with an expected length of ICU stay of more than 48 h was included in the Dutch antibiotic policy guidelines. The results of our study reflect the broad implementation of these policy guidelines. Selective decontamination strategies for preventing VAP were used in 84% of the hospitals and almost two-thirds of Dutch hospitals reported using systemic antibiotics for selective digestive tract decontamination and oropharyngeal decontamination. However, in all Dutch studies on selective digestive tract decontamination in the ICU, evaluation of VAP was performed in a research setting [43]. The remaining VAP prevention practices were only moderately implemented, perhaps as a result of the focus on selective decontamination strategies.

Fourth, infection prevention practices and surveillance systems for monitoring CDI are highly implemented in Dutch hospitals. Soon after CDI outbreaks in the Netherlands in 2005, a national surveillance program for CDI was initiated to rapidly recognize CDI and prevent further spread [44]. Simultaneously, a nationwide multi-modal approach was introduced to prevent CDI, recommending: (a) restricted use of antibiotics; (b) strict enteric precautions when looking after patients with diarrhea; and (c) meticulous cleaning of clinical areas [45]. The well-implemented CDI prevention practices found in this study are therefore in line with our expectations.

Fifth, our data show that hospital-wide antimicrobial stewardship programs have been established in nearly all of the respondent hospitals (91%), corresponding with the findings of Kallen and colleagues on the current state of antimicrobial stewardship in Dutch hospitals [46]. Since 2014, it is mandatory for each hospital in the Netherlands to have an antimicrobial stewardship team, with the task of monitoring the quality of the antibiotic use in their hospital.

Finally, urinary catheters and central venous catheters impregnated with antibiotics or antiseptics are seldom used in the Netherlands. The Netherlands Society of Intensive Care does not advocate for using antimicrobial catheters, based on a recent review concluding that beneficial effects of catheter impregnation on relevant patient outcomes such as clinical sepsis and mortality have not been demonstrated [47].

Our findings should be interpreted in the context of several limitations. First, our response rate was less than 100% and the non-response could have biased our results. Nevertheless, the characteristics of the non-responding hospitals were comparable to those of the 47 hospitals (65%) that participated in our study. We therefore believe that our results generally reflect the use of infection prevention practices by Dutch hospitals. Second, we used self-reported data of infection control professionals. Our findings on some infection prevention practices could be inaccurate if the respondent was not sufficiently aware of all actual practices. An individual respondent may have overestimated or underestimated how frequently the various practices were used [48]. However, we have thoroughly instructed respondents - both verbally and in writing - to consult a colleague if certain infection prevention practices were beyond their scope of attention. We therefore have no reason to believe that systematic inaccuracies have occurred. Still, we cannot rule out potential response bias (e.g. recall, social-desirability) and acknowledge that respondent answers may not correlate perfectly with the actual day-to-day reality of their respective hospital. Third, as our intent was to present a cross-sectional snapshot of infection prevention in Dutch hospitals, our analyses are limited to univariate analyses. As a consequence, inferences regarding the determinants of regular use of the infection prevention practices are not provided. Finally, although we collected a considerable amount of information about the use of infection prevention practices, we do not have data on actual infection rates. As a consequence we were not able to present relationships between the use of the various infection prevention practices and infection outcomes. Nevertheless, research has shown that most of the infection prevention practices included in our study contribute substantially to reducing HAIs; such practices are generally included in published national and international guidelines.

## Conclusion

Our study is, to our knowledge, the first national assessment of HAI prevention practices in the Netherlands. Although many Dutch acute care hospitals are using most of recommended HAI prevention practices, there is currently wide variation among Dutch hospitals in the use of practices focused on preventing CAUTI and VAP. To further improve the adoption of key infection prevention practices among Dutch hospitals, hospital-wide implementation strategies - informed by known barriers and facilitators - are needed. These strategies could include engaging in large-scale collaborative networks that focus attention on HAI prevention and provide guidance and implementation tools to hospitals that may be struggling [49]. Given the success of such collaborative efforts to prevent CLABSI and CDI in Dutch hospitals, we anticipate that similar efforts to prevent CAUTI and VAP will also be successful.

## Supplementary information

**Additional file 1.** TRIP Dutch questionnaire.

## Data Availability

The datasets used and analyzed in the context of this survey are available from the corresponding author upon reasonable request.

## Abbreviations

CAUTI: Catheter-associated urinary tract Infection
CDI: *Clostridioides difficile* infection
CLABSI: Central line-associated bloodstream infection
HAI: Healthcare-associated infection
ICU: Intensive care unit
VAP: Ventilator-associated pneumonia

## References

[1] Stone PW (2009). Economic burden of healthcare-associated infections: an American perspective. Expert Rev Pharmacoecon Outcomes Res.

[2] Klevens RM, Edwards JR, Richards CL, Horan TC, Gaynes RP, Pollock DA (2007). Estimating health care-associated infections and deaths in U.S. hospitals, 2002. Public Health Rep (Washington, DC: 1974).

[3] Burke JP (2003). Infection control - a problem for patient safety. N Engl J Med.

[4] Cassini A, Plachouras D, Eckmanns T, Abu Sin M, Blank HP, Ducomble T (2016). Burden of six healthcare-associated infections on European population health: estimating incidence-based disability-adjusted life years through a population prevalence-based Modelling study. PLoS Med.

[5] Cassini A, Hogberg LD, Plachouras D, Quattrocchi A, Hoxha A, Simonsen GS (2019). Attributable deaths and disability-adjusted life-years caused by infections with antibiotic-resistant bacteria in the EU and the European economic area in 2015: a population-level modelling analysis. Lancet Infect Dis.

[6] Suetens C, Hopkins S, Kolman J, Hogberg L. Point prevalence survey of healthcare-associated infections and antimicrobial use in European acute care hospitals 2011–2012. Stockholm: European Centre for Disease Prevention and Control (ECDC); 2013.

[7] van der Kooi TI, Mannien J, Wille JC, van Benthem BH (2010). Prevalence of nosocomial infections in the Netherlands, 2007-2008: results of the first four national studies. J Hosp Infect.

[8] National Institute for Public Health and the Environment (2018). Surveillance of hospital acquired infections within the Dutch PREZIES network in 2017 [In Dutch: Jaarcijfers 2017: Prevalentieonderzoek ziekenhuizen PREZIES].

[9] Magill SS, Edwards JR, Bamberg W, Beldavs ZG, Dumyati G, Kainer MA (2014). Multistate point-prevalence survey of health care-associated infections. N Engl J Med.

[10] Flodgren G, Conterno LO, Mayhew A, Omar O, Pereira CR, Shepperd S. Interventions to improve professional adherence to guidelines for prevention of device-related infections. Cochrane Database Syst Rev. 2013;(3):Cd006559. 10.1002/14651858.CD006559.pub2 23543545

[11] World Health Organization (2011). Report on the burden of endemic health care-associated infection worldwide.

[12] Umscheid CA, Mitchell MD, Doshi JA, Agarwal R, Williams K, Brennan PJ (2011). Estimating the proportion of healthcare-associated infections that are reasonably preventable and the related mortality and costs. Infect Control Hosp Epidemiol.

[13] Marang-van de Mheen PJ, van Bodegom-Vos L (2016). Meta-analysis of the central line bundle for preventing catheter-related infections: a case study in appraising the evidence in quality improvement. BMJ Qual Saf.

[14] O'Grady NP, Alexander M, Burns LA, Dellinger EP, Garland J, Heard SO (2011). Guidelines for the prevention of intravascular catheter-related infections. Am J Infect Control.

[15] Gould CV, Umscheid CA, Agarwal RK, Kuntz G, Pegues DA (2010). Guideline for prevention of catheter-associated urinary tract infections 2009. Infect Control Hosp Epidemiol.

[16] Marschall J, Mermel LA, Classen D, Arias KM, Podgorny K, Anderson DJ (2008). Strategies to prevent central line-associated bloodstream infections in acute care hospitals. Infect Control Hosp Epidemiol.

[17] Coffin SE, Klompas M, Classen D, Arias KM, Podgorny K, Anderson DJ (2008). Strategies to prevent ventilator-associated pneumonia in acute care hospitals. Infect Control Hosp Epidemiol.

[18] Krein SL, Kowalski CP, Hofer TP, Saint S (2012). Preventing hospital-acquired infections: a national survey of practices reported by U.S. hospitals in 2005 and 2009. J Gen Intern Med.

[19] Saint S, Kowalski CP, Kaufman SR, Hofer TP, Kauffman CA, Olmsted RN (2008). Preventing hospital-acquired urinary tract infection in the United States: a national study. Clin Infect Dis.

[20] Krein SL, Kowalski CP, Damschroder L, Forman J, Kaufman SR, Saint S (2008). Preventing ventilator-associated pneumonia in the United States: a multicenter mixed-methods study. Infect Control Hosp Epidemiol.

[21] Krein SL, Hofer TP, Kowalski CP, Olmsted RN, Kauffman CA, Forman JH (2007). Use of central venous catheter-related bloodstream infection prevention practices by US hospitals. Mayo Clin Proc.

[22] Krein SL, Olmsted RN, Hofer TP, Kowalski C, Forman J, Banaszak-Holl J (2006). Translating infection prevention evidence into practice using quantitative and qualitative research. Am J Infect Control.

[23] Krein SL, Greene MT, Apisarnthanarak A, Sakamoto F, Tokuda Y, Sakihama T (2017). Infection Prevention Practices in Japan, Thailand, and the United States: Results From National Surveys. Clin Infect Dis.

[24] Apisarnthanarak A, Greene MT, Kennedy EH, Khawcharoenporn T, Krein S, Saint S (2012). National survey of practices to prevent healthcare-associated infections in Thailand: the role of safety culture and collaboratives. Infect Control Hosp Epidemiol.

[25] The Health and Youth Care Inspectorate - Ministry of Health WaS (2016). Infection prevention in hospitals should be improved Utrecht, the Netherlands.

[26] Allegranzi B, Pittet D (2008). Preventing infections acquired during health-care delivery. Lancet (London, England).

[27] Saint S, Howell JD, Krein SL (2010). Implementation science: how to jump-start infection prevention. Infect Control Hosp Epidemiol.

[28] Grol R, Wensing M, Eccles M, Davis D (2013). Improving patient care: the implementation of change in health care: John Wiley & Sons.

[29] Saint S, Lipsky BA, Goold SD (2002). Indwelling urinary catheters: a one-point restraint?. Ann Intern Med.

[30] Saint S, Greene MT, Krein SL, Rogers MA, Ratz D, Fowler KE (2016). A program to prevent catheter-associated urinary tract infection in acute care. N Engl J Med.

[31] Meddings J, Rogers MA, Krein SL, Fakih MG, Olmsted RN, Saint S (2014). Reducing unnecessary urinary catheter use and other strategies to prevent catheter-associated urinary tract infection: an integrative review. BMJ Qual Saf.

[32] van der Kooi TI, Wille JC, van Benthem BH (2012). Catheter application, insertion vein and length of ICU stay prior to insertion affect the risk of catheter-related bloodstream infection. J Hosp Infect.

[33] Suetens C (2015). European surveillance of healthcare-associated infections in intensive care units – HAI-Net ICU protocol, version 1.02.

[34] National Institute for Public Health and the Environment (2015). Incidence CLABSI 2005-2014 PREZIES [In Dutch: Referentiecijfers 2005 t/m 2014: Lijnsepsis PREZIES].

[35] National Institute for Public Health and the Environment (2018). Incidence CLABSI 2012-2016 PREZIES [In Dutch: Referentiecijfers 2012 t/m 2016: Lijnsepsis PREZIES].

[36] Baines R, Langelaan M, de Bruijne M, Spreeuwenberg P, Wagner C (2015). How effective are patient safety initiatives? A retrospective patient record review study of changes to patient safety over time. BMJ Qual Saf.

[37] Klopotowska J, Schutijser B, de Bruijne M, Wagner C (2016). Second evaluation of the VMS safety program [In Dutch: Tweede evaluatie van het VMS Veiligheidsprogramma].

[38] Ista E, van der Hoven B, Kornelisse RF, van der Starre C, Vos MC, Boersma E (2016). Effectiveness of insertion and maintenance bundles to prevent central-line-associated bloodstream infections in critically ill patients of all ages: a systematic review and meta-analysis. Lancet Infect Dis.

[39] Klein Klouwenberg PM, van Mourik MS, Ong DS, Horn J, Schultz MJ, Cremer OL (2014). Electronic implementation of a novel surveillance paradigm for ventilator-associated events. Feasibility and validation. Am J Respir Crit Care Med.

[40] Rea-Neto A, Youssef NC, Tuche F, Brunkhorst F, Ranieri VM, Reinhart K (2008). Diagnosis of ventilator-associated pneumonia: a systematic review of the literature. Crit Care (London, England).

[41] Klompas M (2010). Interobserver variability in ventilator-associated pneumonia surveillance. Am J Infect Control.

[42] Oostdijk EAN, Kesecioglu J, Schultz MJ, Visser CE, de Jonge E, van Essen EHR (2014). Effects of decontamination of the oropharynx and intestinal tract on antibiotic resistance in ICUs: a randomized clinical trial. Jama..

[43] Oostdijk E (2015). Selective decontamination in ICU patients: Dutch guideline. Hospital.

[44] Hensgens MP, Goorhuis A, Notermans DW, van Benthem BH, Kuijper EJ. Decrease of hypervirulent *Clostridium difficile* PCR ribotype 027 in the Netherlands. Euro Surveill. 2009;14(45). 10.2807/ese.14.45.19402-en 19941791

[45] Vonberg RP, Kuijper EJ, Wilcox MH, Barbut F, Tull P, Gastmeier P (2008). Infection control measures to limit the spread of Clostridium difficile. Clin Microbiol Infect.

[46] Kallen MC, Ten Oever J, Prins JM, Kullberg BJ, Schouten JA, Hulscher M (2018). A survey on antimicrobial stewardship prerequisites, objectives and improvement strategies: systematic development and nationwide assessment in Dutch acute care hospitals. J Antimicrob Chemother.

[47] Lai NM, Chaiyakunapruk N, Lai NA, O'Riordan E, Pau WS, Saint S (2016). Catheter impregnation, coating or bonding for reducing central venous catheter-related infections in adults. Cochrane Database Syst Rev.

[48] Adams AS, Soumerai SB, Lomas J, Ross-Degnan D (1999). Evidence of self-report bias in assessing adherence to guidelines. Int J Qual Health Care.

[49] Vaughn VM, Saint S, Krein SL, Forman JH, Meddings J, Ameling J (2019). Characteristics of healthcare organisations struggling to improve quality: results from a systematic review of qualitative studies. BMJ Qual Saf.

