# Supplementary material for: Infection prevention practices in the Netherlands: results from a National Survey
Source: Antimicrob Resist Infect Control. 2020 Jan 6;9:7. doi: 10.1186/s13756-019-0667-3 (PMC6945725; doi:10.1186/s13756-019-0667-3)
Supplement: Supplementary file 1 — Additional file 1. TRIP Dutch questionnaire. [file 13756_2019_667_MOESM1_ESM.pdf]

Translating Healthcare-Associated Infection Prevention Research into Practice Survey

The University of Michigan and The Ann Arbor VAMC Translating Infection Prevention Team

Adapted and modified to the Dutch context by the translating infection prevention team of IQ healthcare,
Radboudumc university medical center, the Netherlands

Part 1: Infection-prevention policy en general infection-prevention measures in your hospital

1. In your hospital, are there clear infection-prevention guidelines present that support caregivers in performing infection prevention practices?

- ☐ Yes
- ☐ No → To question 3
- ☐ I don't know → To question 3

2. Are all the resources and materials available to adhere to these infection-prevention guidelines?

- ☐ Yes
- ☐ Partly
- ☐ No
- ☐ I don't know

3. How, in general, do you judge the support of the hospital management (Infection Committee, Board of Directors) infection-prevention policy in your hospital?

- ☐ Very poor
- ☐ Poor
- ☐ Moderate
- ☐ Good
- ☐ Very good
- ☐ I don't know

4. In which department/ organizational unit is your Infection Control Program located?

5. Does incidence measurement of hospital infections take place in your hospital?

- ☐ No
- ☐ Yes, via PREZIES
- ☐ Yes, our own incidence measurement
- ☐ Other (specific situation in this facility): ____

6. Does prevalence measurement for hospital infections take place in your hospital?

- ☐ No
- ☐ Yes, via PREZIES
- ☐ Yes, our own prevalence measurement
- ☐ Other (specific situation in this facility): ____

7. Does your hospital use a system for reporting incidents of hospital infections?

- ☐ No
- ☐ Yes, hospital-wide
- ☐ Yes, on specific wards

8. Does your hospital use a system for analyzing incidents of hospital infections?

- ☐ No
- ☐ Yes, hospital wide
- ☐ Yes, on specific wards

9. Does your hospital give feedback to the wards/healthcare professionals about the results of an incident analysis regarding hospital infections?

- No
- Yes, in writing
- Yes, via intranet
- Yes, during work meetings
- Yes, per individual employee
- Other:

10. Does the hospital organize meetings with nurses and doctors to discuss the surveillance results of hospital associated infections and/or progress of infection prevention initiatives and evaluation?

- Yes, with doctors (not with nurses)
- Yes, with nurses (not with doctors)
- Yes, with nurses and with doctors
- No
- I don't know

11.Does your hospital has a Antimicrobial Stewardship program?

- Yes
- No
- I don't know

12. We would like to ask you to fill in, for each job specification, how many are working in your hospital and what the number of Full-Time Equivalent (FTE) is that they fill jointly:

	Number in your hospital	FTE
Infection-prevention experts		
Medical microbiologists		

13. Does your hospital cooperate with other hospitals or agencies with regard to stimulating infection prevention?

- No
- Yes, the hospital cooperates with:
- Yes, in the future the hospital wants to cooperate with:

14. Please indicate in the table below to what extent you agree or disagree with the statement by ticking the box that is most appropriate.

	Totally agree	Agree	Not agree nor dis-agree	Dis-agree	Totally dis-agree	Not applicable
A. Managers in our hospital take the initiative to ensure that safety is paramount	☐	☐	☐	☐	☐	☐
B. I would feel safe as a patient in our hospital	☐	☐	☐	☐	☐	☐
C. The employees in this hospital are receptive to changes in their work routine	☐	☐	☐	☐	☐	☐
D. The employees in this hospital feel personally responsible for bringing about improvements in care	☐	☐	☐	☐	☐	☐
E. The employees in this hospital are aware of the risks of their own actions	☐	☐	☐	☐	☐	☐
F. This hospital has an open atmosphere in which employees report the incorrect execution of care activities	☐	☐	☐	☐	☐	☐
G. In this hospital there is no time for reflection	☐	☐	☐	☐	☐	☐
H. The employees in this hospital have sufficient skills to adequately apply the recommended infection prevention measures	☐	☐	☐	☐	☐	☐
I. In our hospital it is easy to implement infection prevention measures	☐	☐	☐	☐	☐	☐
J. Participating in patient safety initiatives is important for a good implementation of infection prevention measurements	☐	☐	☐	☐	☐	☐
K. In this hospital, employees are too busy to invest time in new interventions	☐	☐	☐	☐	☐	☐
L. Despite the high workload, employees evaluate their care tasks	☐	☐	☐	☐	☐	☐

15a. The following questions relate to adult patients in your hospital. You will find general infection-prevention measures in the left column.

<u>Can you indicate to what extent the measures are applied in your hospital (where 1 means never and 5 means always)</u>

	1<br>Never	2	3	4	5<br>Always
A. Hand disinfection with alcohol	☐	☐	☐	☐	☐
B. Active surveillance for methicillin-resistant Staphylococcus aureus (MRSA)	☐	☐	☐	☐	☐
C. Patients and employees who are carriers of MRSA immediately undergo measures for skin, hair, and nose disinfection	☐	☐	☐	☐	☐

16b. What is your opinion of the scientific evidence that underlies these general infection-prevention measures.
<u>Please indicate this on the scale below (where 1 means no evidence en 5 means very good evidence)</u>

	1<br>Never	2	3	4	5<br>Always
A. Hand disinfection with alcohol	☐	☐	☐	☐	☐
B. Active surveillance for methicillin-resistant Staphylococcus aureus (MRSA)	☐	☐	☐	☐	☐
C. Patients and employees who are carriers of MRSA immediately undergo measures for skin, hair, and nose disinfection	☐	☐	☐	☐	☐

Part 2: General questions about compliance with infection-prevention bundles

17. Are nurses and doctors required to structurally record their compliance with the infection-prevention bundles (e.g. in patient records)?

- ☐ No, for no infection-prevention bundle
- ☐ Yes, for all infection-prevention bundles
- ☐ Yes, for the following bundles:

18. Has the hospital management or the Board of Directors set targets for optimal compliance with the infection-prevention bundles?

- ☐ No, for no infection-prevention bundle
- ☐ Yes, for all infection-prevention bundles
- ☐ Yes, for the following bundles:

19. Is compliance with the infection-prevention bundles assessed?

- ☐ No, for no infection-prevention bundle
- ☐ Yes, for all infection-prevention bundles
- ☐ Yes, for the following bundles:

20. Is assessed compliance which the infection-prevention reported back to the users?

- ☐ No, for no infection-prevention bundle
- ☐ Yes, for all infection-prevention bundles
- ☐ Yes, for the following bundles:

21. We would like to know how important your hospital managers think it is to prevent the different infections.
<u>Can you indicate on the scale below to what extent the manager of your hospital finds it important to prevent the infections mentioned (where 1 means "very unimportant" and 5 means "very important")</u>

	1 Very unimpor-tant	2	3	4	5 Very impor-tant
A. Preventing catheter-associated urinary tract infections	☐	☐	☐	☐	☐
B. Preventing ventilator-associated pneumonia	☐	☐	☐	☐	☐
C. Preventing central line-associated bloodstream infections	☐	☐	☐	☐	☐
D. Preventing Clostridium difficile infections	☐	☐	☐	☐	☐

Part 3: Catheter-associated urinary tract infection

22a. The following questions relate to adult patients in your hospital. Infection-prevention measures for preventing catheter-related urinary infections are in the left column.
Can you indicate to what extent the measures are applied in your hospital (where 1 means never and 5 means always)

	1<br>Never	2	3	4	5<br>Always
A. Ultrasound methods for bladder residue determination	☐	☐	☐	☐	☐
B. Urinary catheter reminder or stop-order	☐	☐	☐	☐	☐
C. Nurse-initiated urinary catheter discontinuation	☐	☐	☐	☐	☐
D. Nitrofurazone-impregnated catheters	☐	☐	☐	☐	☐
E. Catheters with a silver hydrogel coating (silver-coated catheters)	☐	☐	☐	☐	☐
F. Condom catheters in men	☐	☐	☐	☐	☐
G. Aseptic technique during indwelling urethral catheter insertion and maintenance	☐	☐	☐	☐	☐
H. Intermittent catheterization	☐	☐	☐	☐	☐
I. Suprapubic catheterization	☐	☐	☐	☐	☐

23. What is your opinion about the scientific evidence that underlies these recommended infection-prevention measures.
Please indicate this on the scale below (where 1 means no evidence en 5 means very good evidence)

	1<br>No evidence	2	3	4	5<br>Very good evidence
A. Ultrasound methods for bladder residue determination	☐	☐	☐	☐	☐
B. Urinary catheter reminder or stop-order	☐	☐	☐	☐	☐
C. Nurse-initiated urinary catheter discontinuation	☐	☐	☐	☐	☐
D. Nitrofurazone-impregnated catheters	☐	☐	☐	☐	☐
E. Catheters with a silver hydrogel coating (silver-coated catheters)	☐	☐	☐	☐	☐
F. Condom catheters in men	☐	☐	☐	☐	☐
G. Aseptic technique during indwelling urethral catheter insertion and maintenance	☐	☐	☐	☐	☐
H. Intermittent catheterization	☐	☐	☐	☐	☐
I. Suprapubic catheterization	☐	☐	☐	☐	☐

24. Are patients in your hospital screened for the presence of an urinary tract infection on admission?
- ☐ No
- ☐ Yes
- ☐ I don't know

25.Could you specify which patient groups are screened during their intake for the presence of an urinary tract infection?
- ☐ Frail elderly
- ☐ Children
- ☐ Cancer patients
- ☐ Heart patients
- ☐ Long patients
- ☐ Patients with severe neurological disease

26. Tick the options where placing an indwelling urinary catheter is indicated.
<u>NB: Several situations can be ticked and for the option "other" you can add your own item.</u>
- ☐ Obstruction of the flow of urine from the kidney
- ☐ Urinary incontinence (without obstruction)
- ☐ Monitoring of urine production (for example, of critically ill patients)
- ☐ Peri-operative precautions
- ☐ Reduction of nurses' workload
- ☐ At the request of the patient and/or the family
- ☐ Urinary incontinence of patients with an open sacral or perineal wound
- ☐ Administration of drugs (e.g. amphotericin B) into the bladder
- ☐ Other:

27. Does your hospital use a system to monitor the number of patients with an indwelling urinary catheter?
- ☐ No
- ☐ Yes, hospital-wide
- ☐ Yes, on specific wards

28. Does your hospital use a system to monitor duration and/or discontinuation of indwelling urinary catheters?
- ☐ No
- ☐ Yes, hospital-wide
- ☐ Yes, on specific wards

29. What is the risk (in percentages) of acquiring a catheter-associated urinary tract infection in the departments listed below?
- ☐ Emergency ....%
- ☐ Operating rooms ....%
- ☐ Intensive Care ....%
- ☐ Nursing ward ....%
- ☐ Day treatment ....%
- ☐ Outpatient department........%
- ☐ Other: ....%

30. Does your hospital have an established surveillance system for monitoring urinary tract infection rates?
- ☐ No
- ☐ Yes, hospital-wide
- ☐ Yes, on specific wards

31. Does your hospital report urinary tract infection rates to direct care providers?
- ☐ No
- ☐ Yes, to the whole hospital
- ☐ Yes, on specific wards

Part 4: Central line-associated infection

32a. The following questions relate to adult patients in your hospital. You will find general measures for preventing infections related to central venous catheters in the left column.
<u>Can you indicate to what extent the measures are applied in your hospital (where 1 means never and 5 means always)</u>

	1<br>Never	2	3	4	5<br>Always
A. Maximum sterile barrier precautions (full gown, sterile gloves, full body sterile drape) during central catheter insertion	☐	☐	☐	☐	☐
B. Chlorhexidine gluconate for antisepsis of the insertion site	☐	☐	☐	☐	☐
C. Catheters impregnated with antiseptics	☐	☐	☐	☐	☐
D. Catheters impregnated with antibiotics	☐	☐	☐	☐	☐
E. Routine central catheter changes even if there is no suspicion of a central line infection	☐	☐	☐	☐	☐
F. Antimicrobial dressings	☐	☐	☐	☐	☐
G. Avoidance of the femoral site for line insertions	☐	☐	☐	☐	☐

33b. What is your opinion of the scientific evidence that underlies these recommended infection-prevention measures.
<u>Please indicate this on the scale below (where 1 means no evidence en 5 means very good evidence)</u>

	1<br>Never	2	3	4	5<br>Always
H. Maximum sterile barrier precautions (full gown, sterile gloves, full body sterile drape) during central catheter insertion	☐	☐	☐	☐	☐
I. Chlorhexidine gluconate for antisepsis of the insertion site	☐	☐	☐	☐	☐
J. Catheters impregnated with antiseptics	☐	☐	☐	☐	☐
K. Catheters impregnated with antibiotics	☐	☐	☐	☐	☐
L. Routine central catheter changes even if there is no suspicion of a central line infection	☐	☐	☐	☐	☐
M. Antimicrobial dressings	☐	☐	☐	☐	☐
N. Avoidance of the femoral site for line insertions	☐	☐	☐	☐	☐

34. Who is responsible for inserting the majority of the central venous catheters?
- ☐ Resident
- ☐ Radiologist
- ☐ Anesthesiologist
- ☐ Intensivist
- ☐ Internist
- ☐ Surgeon

35. Does your hospital use standardized kits or carts for central venous catheter insertion?
- ☐ No
- ☐ Yes, hospital-wide
- ☐ Yes, on specific wards

36.Does your hospital has a procedure to assess the eligibility of a central venous catheter before it is inserted?
- ☐ No
- ☐ Yes, hospital-wide
- ☐ Yes, on specific wards

37. In your hospital, is there a daily check whether the presence of a central venous catheter is still indicated?
- ☐ No
- ☐ Yes, hospital-wide
- ☐ Yes, on specific wards

38. What is the risk (in percentages) of acquiring a central line-associated bloodstream infection in the departments listed below?
- ☐ Emergency ……..%
- ☐ Operating rooms ……..%
- ☐ Intensive Care ……..%
- ☐ Nursing ward ……..%
- ☐ Day treatment ……..%
- ☐ Outpatient department……..%
- ☐ Other: ……..%

39. Does your hospital have an established surveillance system for monitoring central line-associated infection rates?
- ☐ No
- ☐ Yes, hospital-wide
- ☐ Yes, on specific wards

40. Does your hospital report central line-associated infection rates to direct care providers?
- ☐ No
- ☐ Yes, to the whole hospital
- ☐ Yes, to specific wards

Part 5: Ventilator-associated pneumonia

41a. The following questions relate to adult patients in your hospital. In the left column, you will find infection-prevention measures for preventing ventilator-associated pneumonia.
<u>Can you indicate to what extent the measures are applied in your hospital (where 1 means never and 5 means always)</u>

	1 Never	2	3	4	5 Always
A. Semi-recumbent positioning of the patient (head of bed elevated 30 degrees or more)	☐	☐	☐	☐	☐
B. Antimicrobial mouth rinse	☐	☐	☐	☐	☐
C. Subglottic secretion drainage	☐	☐	☐	☐	☐
D. Oscillating/ kinetic beds	☐	☐	☐	☐	☐
E. Topical and/or systemic antibiotics for selective digestive tract decontamination (SDD)	☐	☐	☐	☐	☐
F. Oropharyngeal decontamination with no standard intravenous prophylaxis (SOD)	☐	☐	☐	☐	☐
G. Silver-coated endotracheal tube	☐	☐	☐	☐	☐
H. “Sedation vacation” (e.g., regular interruption of sedation)	☐	☐	☐	☐	☐

42b. What is your opinion of the scientific evidence that underlies these recommended infection-prevention measures.

<u>Please indicate this on the scale below (where 1 means no evidence en 5 means very good evidence)</u>

	1 No evidence	2	3	4	5 Very good evidence
A. Semi-recumbent positioning of the patient (head of bed elevated 30 degrees or more)	☐	☐	☐	☐	☐
B. Antimicrobial mouth rinse	☐	☐	☐	☐	☐
C. Subglottic secretion drainage	☐	☐	☐	☐	☐
D. Oscillating/ kinetic beds	☐	☐	☐	☐	☐
E. Topical and/or systemic antibiotics for selective digestive tract decontamination (SDD)	☐	☐	☐	☐	☐
F. Oropharyngeal decontamination with no standard intravenous prophylaxis (SOD)	☐	☐	☐	☐	☐
G. Silver-coated endotracheal tube	☐	☐	☐	☐	☐
H. “Sedation vacation” (e.g., regular interruption of sedation)	☐	☐	☐	☐	☐

43. Does your hospital encourage early mobilization of ventilated patients as a strategy to prevent ventilator-associated pneumonia?

- ☐ No
- ☐ Yes, in the whole hospital
- ☐ Yes, on specific wards

44. Does your hospital have an established surveillance system for monitoring ventilator-associated pneumonia rates

- ☐ No
- ☐ Yes, hospital-wide
- ☐ Yes, on specific wards

45. Does your hospital report ventilator-associated pneumonia rates to direct care providers?

- ☐ No
- ☐ Yes, to the whole hospital
- ☐ Yes, to specific wards

Part 6: Clostridium difficile infection

46a. The following questions relate to adult patients in your hospital. You will find infection-prevention measures for *Clostridium difficile* infections preventing ventilation-related pneumonia in the left column. Can you indicate to what extent the measures are applied in your hospital (where 1 means never and 5 means always)

	1 Never	2	3	4	5 Always
A. Aseptic techniques (gloves and gowns) for employees who care for patients with a C. difficle infection for duration of diarrhea	☐	☐	☐	☐	☐
B. Single room with private bathroom for the patient with diarrhea that (presumably) is caused by a C. difficile infection	☐	☐	☐	☐	☐
C. Apply hand hygiene by means of soap and water after entering the room of a patient infected with C. difficile	☐	☐	☐	☐	☐
D. Thorough cleaning and disinfection (with a cleaning product containing chlorine bleach) of the room and the materials with which the patient is cared for	☐	☐	☐	☐	☐
E. Daily cleaning of the surfaces that are most touched (edge of the bed, light switch, doorknob, etc.).	☐	☐	☐	☐	☐
F. Disposable thermometers for patients infected with C. difficile	☐	☐	☐	☐	☐

47b. What is your opinion of the scientific evidence that underlies these recommended infection-prevention measures.
Please indicate this on the scale below (where 1 means no evidence en 5 means very good evidence)

	1 No evidence	2	3	4	5 Very good evidence
A. Aseptic techniques (gloves and gowns) for employees who care for patients with a C. difficle infection for duration of diarrhea	☐	☐	☐	☐	☐
B. Single room with private bathroom for the patient with diarrhea that (presumably) is caused by a C. difficile infection	☐	☐	☐	☐	☐
C. Apply hand hygiene by means of soap and water after entering the room of a patient infected with C. difficile	☐	☐	☐	☐	☐
D. Thorough cleaning and disinfection (with a cleaning product containing chlorine bleach) of the room and the materials with which the patient is cared for	☐	☐	☐	☐	☐
E. Daily cleaning of the surfaces that are most touched (edge of the bed, light switch, doorknob, etc.).	☐	☐	☐	☐	☐
F. Disposable thermometers for patients infected with C. difficile	☐	☐	☐	☐	☐

48.Do healthcare professionals in your hospital receive training on which symptoms the patient should be tested for C. difficile?

- ☐ No
- ☐ Yes
- ☐ I don’t know

49. Does your hospital have a protocol for routinely testing for C. difficile as soon as patients develop diarrhea?

- ☐ No
- ☐ Yes, for the whole hospital
- ☐ Yes, for specific wards

50. Does your hospital have an established surveillance system for monitoring the number of patients with C. difficile infection?

- ☐ No
- ☐ Yes, hospital-wide
- ☐ Yes, for specific wards

51. Does your hospital report C. difficile infection rates to direct care providers?

- ☐ No
- ☐ Yes, to the whole hospital
- ☐ Yes, to specific wards

Part 7: Concluding questions

52.What is the total number of acute care beds in your hospital?

53.What is the total number of Intensive Care (ICU) beds in your hospital?

54.What is the percentage for the distribution of rooms?

- Single bed rooms: %
- Two or multiple bed rooms: %

55. Specify your function below

56. Please specify your professional certifications and educational degrees:

57. How long have you been working in this position?

58. How long have you been working at this hospital?
